# NOTCH1 Intracellular Domain and the Tumor Microenvironment as Prognostic Markers in HNSCC

**DOI:** 10.3390/cancers14041080

**Published:** 2022-02-21

**Authors:** Benedikt Schmidl, Michael Siegl, Melanie Boxberg, Fabian Stögbauer, Daniel Jira, Christof Winter, Leonhard Stark, Anja Pickhard, Barbara Wollenberg, Markus Wirth

**Affiliations:** 1 Department of Otolaryngology Head and Neck Surgery, Technical University Munich, 81675 Munich, Germany; benedikt.schmidl@tum.de (B.S.); michael.siegl@tum.de (M.S.); daniel.jira@tum.de (D.J.); leo.stark@tum.de (L.S.); anja.pickhard@tum.de (A.P.); barbara.wollenberg@tum.de (B.W.); 2 Institute of Pathology, Technical University Munich, 81675 Munich, Germany; melanie.boxberg@tum.de (M.B.); fabian.stoegbauer@tum.de (F.S.); 3 Institute of Clinical Chemistry and Pathobiochemistry, Technical University Munich, 81675 Munich, Germany; christof.winter@tum.de

**Keywords:** HNSCC, NOTCH1, immunoscore, immune system, HPV, NICD, OPSCC

## Abstract

**Simple Summary:**

In the head and neck, a large proportion of squamous cell carcinoma demonstrate a mutation of the NOTCH1 gene. The aim of this project was to investigate the role of NOTCH1 and immunological characteristics and highlight a potential rationale for therapy. We found that a high expression of NOTCH1 intracellular domain in these patients is associated with reduced overall survival. In vitro experiments additionally showed a reduction of migration and proliferation of cancer cells when NOTCH1 was knocked down. NOTCH1 is, therefore, most likely involved in migration and proliferation of head and neck squamous cell carcinoma and is a prognostic marker in these patients.

**Abstract:**

(1) Background: NOTCH1 is the second most common mutated gene in whole-exome sequencing of HNSCC. The aim of this project was to gain further insight into the relevance of NOTCH1 in HNSCC, potentially establishing NOTCH1 as a prognostic marker or therapeutic target; (2) Methods: NOTCH1 was silenced via RNA interference in six HNSCC cell lines and the impact was evaluated in migration and proliferation assays. Subsequently, the protein expression of NOTCH1 intracellular domain (NICD) and NOTCH1 mRNA expression were examined in 70 oropharyngeal squamous cell cancer tissue samples. Lastly, the NICD expression was compared with the local infiltration of lymphocytes, measured with the immunoscore; (3) Results: Knockdown of NOTCH1 decreased migration and proliferation. A high NICD expression was associated with lower OS. A high immunoscore resulted in significantly better OS. NICD expression was independent of the immunoscore and as a whole differentiated three distinct prognostic groups; (4) Conclusions: These data suggest that NOTCH1 is involved in migration and proliferation of HNSCC cell lines. In vivo, NICD expression was associated with overall survival and could, therefore, be used as a prognostic marker. NICD expression differs from NOTCH1 mRNA levels, potentially explaining the previously suggested bimodal role as an oncogene and tumor suppressor in HNSCC.

## 1. Introduction

With more than 650,000 cases each year, head and neck cancer (HNC) are one of the most common cancer entities worldwide [1]. While the most frequent risk factors remain alcohol and tobacco use [2,3], the incidence of a subgroup of oropharyngeal cancer caused by high-risk human papillomavirus (HPV) infection is increasing steadily. HPV-negative and -positive HNSCC differ greatly in prognosis, mutational burden, and molecular pathogenesis. Conventional therapy involves surgery and radio/chemotherapy, with a substantial rate of recurrence [4]. Patients with a recurring tumor have a poor median OS [5]. The need to improve treatment in these patients is hindered by the heterogeneous nature of HNSCC. Finding a molecular target for systemic therapy or as a new prognostic marker remains a challenging task [6,7]. Even though NOTCH1 has been identified as one of the most commonly mutated genes in HNSCC, the discussion about its role is still controversial.

Discovered in D. melanogaster by Thomas Morgan in 1913, NOTCH1 and its highly conserved pathway were found to be involved in mammalian embryonic development of the heart, liver, eye, face, kidney, and vascular system [8]. Either a jagged or delta-like ligand binds to the transmembrane cell receptor, which results in cleavage by gamma-secretase and intracellular release of NOTCH1 intracellular domain (NICD), which then translocates to the nucleus [9] leading to the transcriptional activation of downstream genes, such as HES, HEY1, HEY2, HEYL, MYC, BCL-1, and p21 [10,11].

In recent decades, NOTCH1 has been associated with carcinoma of the pancreas, prostate, breast, and lungs [11,12]. In 2011, NOTCH1 was the second most common mutated gene (after TP53) in whole-exome sequencing of HNSCC, showing mainly inactivating mutations [13,14]. At the same time, NOTCH1 is reported to be overexpressed in a variety of tumors [11] and associated with advanced tumor stages. Several publications suggested that NOTCH1 has a bimodal role in HNSCC, therefore acting as a tumor suppressor and oncogene depending on the context [10,11].

NOTCH1 signaling shapes the tumor immunity in several cancer entities. NOTCH1 is, e.g., important in the regulation of CD8+ T cells and the differentiation of tumor-associated macrophages (TAM) [15]. The association between NOTCH1 signaling in tumor cells and the state of the surrounding immune microenvironment has not been examined so far in HNSCC. The aim of this study was to shed light on the role of NOTCH1 in vitro in HPV-positive and -negative HNSCC cell lines and correlate the impact of activated NOTCH on survival of HNSCC patients. Additionally, a potential association with NOTCH1 expression, HPV status, and infiltrating lymphocytes was assessed.

## 2. Materials and Methods

### 2.1. Cell Culture and Transfection

The 93-VU-147T cell line was obtained from VU University Medical Center Amsterdam (Department for Clinical Genetics, Amsterdam, the Netherlands), UPCI-SCC-111 and -154 cell lines were obtained from DMSZ (Braunschweig, Germany), and UD-SCC-2, -5, and -7 cell lines were obtained from the University of Düsseldorf (Düsseldorf, Germany). All squamous cell carcinoma cell lines were cultured in appropriate medium (DMEM, MEM, or RPMI, Invitrogen, Darmstadt, Germany), supplemented with 10% fetal bovine serum (FBS, Gibco) and 100 μg/mL penicillin and 100 μg/mL streptomycin (Sigma Aldrich, St. Louis, MO, USA), and stored in an incubator (37 °C, 5% CO_2_).

### 2.2. siRNA-Mediated Knockdown

For RNA interference, an artificial siRNA targeting NOTCH1 (Sequence: CCGCCTTTGTGCTTCTGTTCTTCGT) was used at a concentration of 40 nM. Transfection with Oligofectamine (Fisher Scientific, Waltham, MA, USA) was performed according to the manufacturer’s instructions. To test adverse effects of siRNA, scrambled siRNA was initially tested. Cells were harvested 48 h after transfection and were either used for Western blot or consecutive experiments. Full Western blot images can be found at Files S1.

### 2.3. Western Blot

For protein extraction, adherent cells were washed with cold PBS and directly treated with cell lysis buffer (Cell Signaling, Danvers, MA, USA), freshly supplemented with 1 mM PMSF (Carl Roth, Karlsruhe, Germany) and a protease inhibitor cocktail (Cell Signaling, Danvers, MA, USA). Cells were scraped off the culture dish, incubated on ice, and centrifuged for 15 min. The Bradford assay, SDS-Page, semi dry transfer onto a polyvinylidene difluoride (PVDF) membrane, blocking, and incubation with the primary antibody were performed as described by Koch [16]. Primary antibodies against NOTCH1 (1:1000, NOTCH1 D1E11, Cell Signaling Technology, Inc., Danvers, MA, USA) or β-Tubulin antibody (1:1000, 2146, Cell Signaling Technology, Inc., Danvers, MA, USA) were used. After incubation, the membranes were washed in TBS-T and Anti-Rabbit IgG HRP-linked antibody (1:5000, Cell Signaling Technology, Inc., Danvers, MA, USA) was applied for another hour at 4 °C. The membranes were incubated with Thermo Scientific™ Pierce™ ECL Western Blotting Substrate (Fisher Scientific, Waltham, MA, USA) and chemiluminescence detection was performed with the ChemiDoc XRS+ Imager (Bio-Rad Laboratories GmbH, Munich, Germany). Analysis and editing were performed with Image Lab 6 (Bio-Rad Laboratories GmbH, Munich, Germany).

### 2.4. DAPT and DLL4 Treatment

DAPT (Sigma Aldrich, St. Louis, MO, USA) was added to cells in a final concentration of 25 μM. Forty-eight hours after this step, cells were analyzed or used for consecutive experiments. DLL4 (R & D Systems, Minneapolis, MN, USA) was added at a concentration of 2 μg/mL to wells precoated the day before with 0.1% gelatin (Sigma Aldrich, St. Louis, MO, USA). Twenty-four hours after this step, cells were added.

### 2.5. Migration Assay

At time point 0 h, an artificial scratch was made into a confluent monolayer of cells with the tip of a sterile plastic pipette and a ruler. The scratch was washed two times with medium and a picture was taken immediately thereafter. At time point 9 h, an additional picture was taken to trace the migration into the artificial scratch. Adobe Photoshop CS5 was used to calculate the area of the scratch by automatic counting of pixels between the two frontlines of cells. The area was compared to the initial area at time point 0 h after 9 h, using the formula: ((At 0 h – At 9 h)/At 0 h) ∗ 100.

### 2.6. Proliferation Assay

Into each of two 24 well plates, 5 × 10^4^ cells were seeded in triplicates. After 24 h, the first plate was analyzed (timepoint 0 h). Shortly, medium was aspirated, cells were fixed with 4% formaldehyde for 10 min and washed with PBS before staining with Crystal Violet solution (Sigma Aldrich, St. Louis, MO, USA) for 20 min. Subsequently, cells were washed thoroughly three times with aqua distillate and lysed with 1 mL of 1% SDS (Carl Roth GmbH + Co. KG, Karlsruhe, Germany) for 30 min. An aliquot was pipetted into the well of a 96 well plate and the absorbance was measured with a multiwell plate reader (Multiskan FC, Thermo Scientific, Waltham, MA, USA). Blank corrected absorbance at 595 nm served as a surrogate for the number of cells. After 72 h, the second plate was analyzed as described before. The ratio between 72 h and 0 h was used as a measure of change in proliferation.

### 2.7. Patient Cohort

Seventy patients with primary oropharyngeal squamous cell carcinomas, who were treated between January 2008 and June 2011 at the ENT department of the Klinikum rechts der Isar of the Technical University of Munich, were included in this study. Formalin-fixed and paraffin-embedded tissue was collected. The clinical and pathological data was followed up retrospectively by using data from the electronic medical records. The date of surgery to the date of death was used to calculate overall survival. According to the criteria of the Union for International Cancer Control, the TNM staging system (7th edition) was applied. The age of patients ranged from 37 to 79 years (median age was 58.4 years). Kaplan–Meier survival analysis revealed a median survival of 4.22 years. The follow-up period ranged from 0.05 years to 9.00 years. Ethical approval was obtained from the ethics committee of the Technical University of Munich (reference number 13/20S).

### 2.8. Immunohistochemistry

HNSCC were fixed in 10% buffered formalin and embedded in paraffin. With the microtome, 2–3 μm thick sections were cut with the Microm^HM^ 355 S (International GmbH, Walldorf, Germany). Afterwards, sections were deparaffinized and stained with a VENTANA BenchMark GX with NICD antibody (N5375-09A, 1:400, US Biological, Salem, MA, USA). After staining, the sections were dehydrated by immersion in ethanol series and xylol (2 min each) and air dried. In addition, HE staining was conducted manually. For antibody validation, the expression in lymphoid tissue (appendix and tonsil tissue) was used as a control according to the manufacturer’s instructions. A representative image is depicted in Supplementary Figure S1. The presence of a tumor was examined, and three high power fields (HPFs) of each section with the strongest staining were chosen. The intensity of intracellular NICD expression was rated from 0 to 3 (0 = negative, 1 = weak, 2 = moderate, 3 = strong expression). Sections with a level of expression ≥2 were designated as high NICD expressions.

### 2.9. Immunoscore

Staining for CD3 (1:400, Cell Marque, Rocklin, CA, USA), CD8 (1:25, Thermo Fisher Scientific, IL, USA), and p16^INK4a^ (CINTec Roche, Switzerland) was performed with VENTANA BenchMark GX as described above. CD3 and CD8 were used to calculate the immunoscore, a tool for measuring interactions of the tumor margin and the surrounding stromal tissue originally established in colorectal carcinoma [17]. Three HPFs of the tumor core and three HPFs of the invasive margin, the region between malignant cells and peritumoral stroma, were selected and the number of positive stained T lymphocytes in an area of 250 µm^2^ were counted manually. The HPFs with the strongest infiltration were selected. The invasive margin was defined as the area ranging from 50 μm within the tumor to 300 μm outside of it [18]. The median number of lymphocytes in each area were determined for all samples, and each area was classified as high or low infiltration according to the median. Then, the immunoscore was determined according to the guidelines of the task force establishing it in colorectal cancer [19]. The expression of p16^INK4a^ was assessed according to the manufacturer’s guidelines.

### 2.10. RNA Isolation and cDNA Synthesis

From the FFPE OPSCC cohort, 10 µm thick sections were cut and deparaffinized using 200 µL of deparaffinization solution (Qiagen, Hilden, Germany). RNA was extracted with the DNA/RNA FFPE Kit (Qiagen, Hilden, Germany), according to the manufacturer’s protocol. Using a NanoDrop 1000 system (PEQLAP, Erlangen, Germany), the total concentration of RNA was measured. A total of 250 ng of RNA was converted into cDNA using M-MLV-Reverse Transcriptase (Fisher Scientific, Waltham, MA, USA). Due to material consumption and low RNA yield, only 60 FFPE samples could be used.

### 2.11. Quantitative Real-Time PCR

Quantitative real-time PCR was used to quantify mRNA expression of NOTCH1 and GAPDH. A total of 50 ng of cDNA template was added to KAPA SYBR FAST qPCR master Mix (PeqLab, Erlangen, Germany), forward primer, reverse primer, and nuclease-free water. The annealing temperatures and primer sequences are depicted in Table 1. GAPDH was used for normalization and relative expression of NOTCH1 was calculated using the ΔΔCt method.

### 2.12. Statistical Analysis

All in vitro experiments were performed a minimum of three times. Using the Shapiro–Wilk test, the results were tested for normal distribution. Ordinary one-way ANOVA and Dunnet’s multiple comparisons were used to calculate significance. Kaplan–Meier survival analysis was used to compare survival rates for different patient groups and clinical characteristics. To examine the association of NICD expression, immunoscore, and p16 staining with overall survival, multivariate forward stepwise Cox regression was performed. Associations were tested with Fisher’s exact test. At *p* < 0.05, the null hypothesis was rejected, and the result was considered statistically significant. Statistical calculation was performed using Prism 8 (GraphPad Software, La Jolla, CA, USA) and SPSS Statistics for Windows (IBM, Armonk, NY, USA).

## 3. Results

### 3.1. NOTCH1 Is Involved in Migration and Proliferation of HNSCC Cell Lines

The effect of siRNA-mediated NOTCH1 knockdown, inhibition of NOTCH1 signaling via DAPT and stimulation with DLL4 on migration and proliferation was examined in three HPV– and three HPV+ HNSCC cell lines (UD-SCC-5 (UD5), UD-SCC-7 (UD7), UPCI-SCC-111 (UP111), UD-SCC-2 (UD2), UPCI-SCC-154 (UP154), and 93-VU-147T (93VU)).

**Figure 1:**
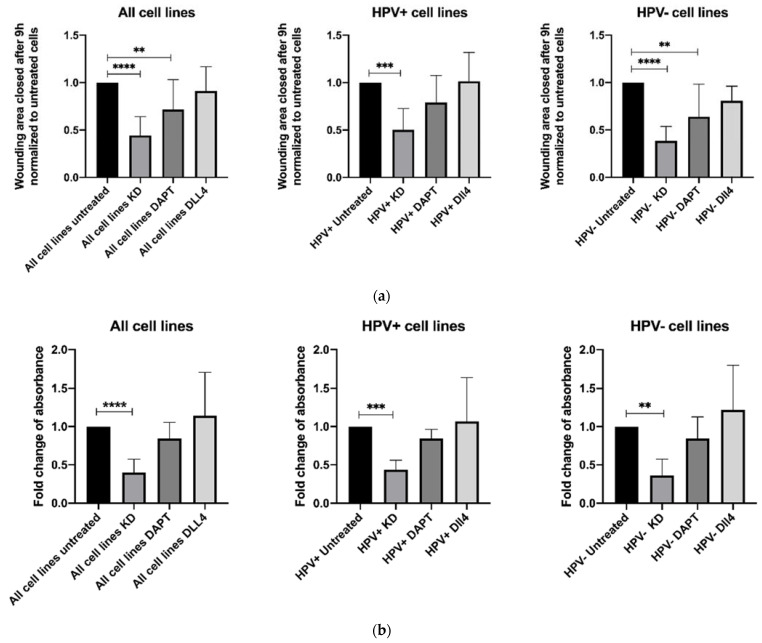
Results of (**a**) migration assay (scratch assay, 9 h) and (**b**) proliferation assay (72 h) after siRNA-mediated NOTCH1 knockdown, inhibition of NOTCH1 signaling via DAPT, and stimulation with DLL4; depicted are the grouped results of all six cell lines, the three HPV+ cell lines and the three HPV− cell lines. Data are depicted as mean ± SD. Significance was tested with one-way ANOVA and Dunnet’s multiple testing (** means *p* < 0.01, *** means *p* < 0.001, **** means *p* < 0.0001).

Overall, the knockdown reduced migration by 55.7% (*p* < 0.0001, Figure 1a). The effect was stronger in HPV– cell lines. Treating cells with DAPT decreased migration significantly in the grouped analysis of all cell lines, and in HPV– cell lines (*p* = 0.0010, *p* = 0.0019, Figure 1a). Treatment with DLL4 did not change migration significantly (Figure 1a). The siRNA-mediated knockdown of NOTCH1 reduced the migration of cells more effectively than DAPT treatment. The detailed effect in each cell line is provided in the Supplementary Materials.

A proliferation assay was used to determine the impact of the inhibition or stimulation of NOTCH1 on proliferation. Knockdown of NOTCH1 resulted in significantly decreased proliferation (*p* < 0.0001, Figure 1b). The effect was stronger in HPV− cell lines. DAPT and DLL4 treatment did not affect proliferation significantly (Figure 1b and Supplementary Table S1). Again, the siRNA-mediated knockdown proved to be more effective than DAPT treatment.

### 3.2. Association between NOTCH1 and NICD Expression and Clinical and Pathological Parameters

The clinical and pathological characteristics of the cohort are depicted in Table 2. Patients with an OPSCC were grouped into high or low NICD IHC and NOTCH1 mRNA expression based on the median. An association between expression and clinical parameters was analyzed.

**Figure 2:**
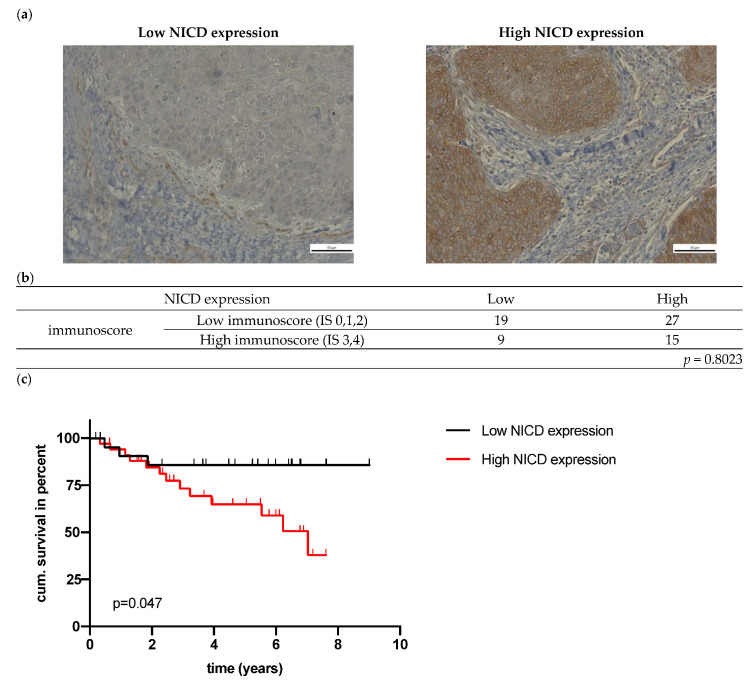

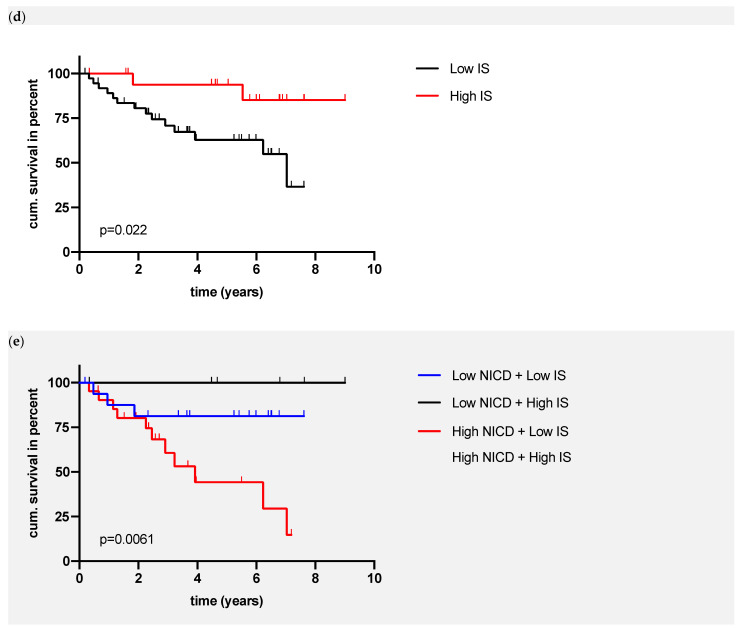
(**a**) Exemplary images of immunohistochemical NICD staining in HNSCC; (**b**) Association of NICD and immunoscore; (**c**) Overall survival of patients in relation to NICD expression (*p* value (log-rank) = 0.0477); (**d**) OS of patients in relation to the immunoscore (*p* value (log-rank) = 0.022); (**e**) OS of patients in relation to NICD expression and the immunoscore (*p* value (log-rank) = 0.0061).

All oropharyngeal SCC tumors were immunohistochemically NICD-positive, 28 had a low expression, and 42 had a high expression (exemplary pictures presented in Figure 2a). NICD expression was significantly higher in p16-positive tumors (Table 2). High NOTCH1 mRNA expression was associated with higher T stage (Table 2). High NICD protein expression, but not NOTCH1 mRNA expression was associated with lower OS (*p* = 0.0477 and *p* = 0.7067) (Figure 2c, Supplementary Figure S1). p16-negative OPSCC with high NICD expression had a lower OS (*p* = 0.0508); this effect was not seen in p16-positive OPSCC (Supplementary Figure S1). No statistically significant association was detected between NICD IHC and NOTCH1 mRNA expression (*p* = 0.1923, Supplementary Figure S1).

Next, the local infiltration with T lymphocytes was assessed with the immunoscore, which is based on CD3 and CD8 infiltration in the core of the tumor and invasive margin. The median number of tumor infiltrating lymphocytes is depicted in Supplementary Figure S1. A high immunoscore, which is equal to a high T cell immunological response, was associated with a significantly better OS (*p* = 0.0220, Figure 2d). p16-positive OPSCC showed a significantly higher number of infiltrating T lymphocytes (*p* = 0.0406, Supplementary Figure S1). The multivariate cox regression confirmed the significant association between the immunoscore and OS (*p* = 0.020); p16 and NICD were not significant (*p* = 0.087, *p* = 0.054). By combining the data of immunohistochemical NICD expression and *immunoscore* (IS), patients can be grouped into four different groups (high NICD + high IS, high NICD + low IS, low IS + high NICD, low IS + low NICD) with a significantly lower survival in patients with high NICD + low IS (*p* = 0.0061, Figure 2e). Based on the Kaplan–Meier curve, three distinct prognostic groups can be differentiated visually (Figure 2e):

- High-risk patients: high NICD and low IS;
- Intermediate-risk patients: low NICD and high IS or high NICD and low IS;
- Low-risk patients: low NICD and high IS.

The five-year survival of the 17 patients in the high-risk group was 44.3%, while none of the patients died in the low-risk group (low NICD + high IS). NICD expression was not associated with the infiltration of lymphocytes (*p* = 0.8023, Figure 2b).

## 4. Discussion

In this study, the functional impact of knockdown, inhibition, and stimulation of the NOTCH1 signaling pathways was assessed in three HPV+ and three HPV− HNSCC cell lines. In addition, the NOTCH1 mRNA and NICD expression was examined in an OPSCC patient cohort. This study marks the first time the NOTCH1/NICD expression was compared with the proportion of tumor infiltrating lymphocytes.

To assess the effect of NOTCH1 signaling, a siRNA-mediated knockdown of NOTCH1 was performed. This knockdown resulted in a significant reduction of migration and proliferation in HPV-positive and -negative cells. Using a similar mechanism, Jing et al. downregulated NOTCH1 signaling via silencing of PDK1, which normally binds to NICD and inhibits its ubiquitin-mediated degradation. Consistent with our results, this has led to a decrease in migration, proliferation, and invasion of FaDu cells [20]. Cancer stem cells, which are associated with aggressive tumor growth and progression in HNSCC, showed a significantly higher expression of NOTCH1 [21]. Zhang et al. analyzed chemosensitivity before chemotherapy treatment with either Paclitaxel, Cisplatin, or 5-FU, and found a strong resistance of fresh HNSCC tissue with high immunohistochemical expression of NOTCH1. Treatment with DAPT increased the response to chemotherapy. On the other hand, deletion of the NOTCH1 gene in murine keratinocytes led to increased proliferation and aberrant differentiation [22]. This is supported by a genomic landscape dominated by loss of function mutations [23]. As a potential explanation for this controversial finding, it was suggested that NOTCH1 acts as an oncogene only in a subgroup of HNSCC [10,23].

Corresponding to the successful DAPT therapy in a murine breast cancer model [24], treatment with DAPT decreased the migratory potential of HNSCC cells in this study, but this effect was not as strong as the siRNA-mediated NOTCH1 knockdown. siRNA-based therapeutics could address this disadvantage in the future [25]. In the subgroup analysis, DAPT inhibited migration significantly only in the HPV-negative cells. As many studies found HPV-negative HNSCC to be more resistant to treatment [10], NOTCH1 may be an attractive target only in HPV-negative HNSCC. In agreement with our results, treatment with DAPT increased radio- and chemosensitivity in vitro and decreased the size of xenograft HNSCC in mice [26,27]. Overall, stimulation of the NOTCH1 pathway via DLL4 did not influence migration and proliferation significantly in the presented study. In breast cancer, a high expression of DLL4 at mRNA and protein levels is associated with advanced tumor stages and poor differentiation [28]. Blockage of DLL4 in an orthotopic mouse model of ovarian cancer reduced tumor weights up to 82% [29]. Non-canonical activation of the NOTCH1 receptor plays an important role in cancer and could explain the weak effect of y-secretase inhibition and DLL4 treatment [30].

Next, to investigate the prognostic role of NOTCH1, NICD protein and NOTCH1 mRNA expression were examined in an OPSCC patient cohort. A significantly better overall survival rate was seen in patients with a low NICD protein expression. A high NICD expression was observed in various cancer tissues and is in line with the findings of this study [10,31,32]. The expression of NICD was first examined by Rettig et al. in 2015, and two distinct patterns of expression were described in OPSCC: a peripheral and a diffuse one. All of their OPSCC stained positive for NICD, and it was suggested that the different staining is a sign of different roles of NOTCH signaling [10]. In this study, NOTCH1 mRNA expression was compared to NICD expression to obtain further insight into the difference between mRNA receptor expression and signaling. Eukaryotic mRNA can be subject to more than 100 different chemical modifications, and new studies shed light on complex mechanisms of gene expression regulation [33]. Assessing both mRNA and protein levels enables a more in-depth look at this potential explanation for the discrepancy of the role of NOTCH1 expression in the literature. In this study, NOTCH1 mRNA expression was associated with higher T-status, but no significant association with NICD expression was detected, suggesting differences between expression and activation of the receptor. There is an ongoing discussion in the literature whether NOTCH1 is an oncogene or tumor suppressor in HNSCC [6,21]. For this reason, the IHC antibody used in this study selectively stained NICD as a surrogate for NOTCH signaling, whereas other studies detected full length NOTCH1 [11,34,35]. This could contribute to explaining differing results in previous studies. Additionally, in this study, only the well-defined HNSCC subset of oropharyngeal squamous cell carcinoma was selected. Staining a larger cohort in a future study with an antibody for full length NOTCH1 and a separate antibody for the extracellular domain may in addition reveal patterns of activation that we did not analyze.

Because NOTCH1 signaling is involved in the regulation of the vascular architecture of the TME, activation of CD8^+^ cytotoxic T cells as well as guiding differentiation of tumor-associated macrophages (TAMs) [15,34,36], interactions of the immune system and NOTCH1 were investigated with the immunoscore, which is based on the infiltration of CD3+ and CD8+ T lymphocytes in the core and the invasive margin of the tumor. The score was initially established for colorectal cancer, to estimate the risk of recurrence and predict the response to treatment [17,36]. A significant association of the immunoscore and OS in OPSCC was discovered in this study. In a similar approach, Ward et al. also demonstrated that the number of tumor-infiltrating lymphocytes predicted outcomes in HPV-positive oropharyngeal cancers [37]. A high infiltration rate of CD8^+^ lymphocytes in HPV-positive HNSCC is associated with a better response to a PD-1 inhibitor [38,39]. The role of the immune system has gained importance since the new group of checkpoint inhibitors has improved the therapy of metastatic or unresectable HNSCC. Pembrolizumab and Nivolumab are able to enhance immune system activity, leading to eradication of cancer cells with less severe side effects than conventional chemotherapy [15,16]. Even though the results are promising, there are still a large group of non-responders [5,40,41]. The non-responders could benefit from combination therapies or alternative therapies, such as NOTCH1-targeted therapies. Thus, an association between NICD expression and the local tumor immune status was assessed for the first time in OPSCC, and no significant association was identified. Non-inflamed or immune-excluded OPSCC could, therefore, be good candidates for NOTCH1-targeted therapy. The next step towards the clinical application of NOTCH targeted therapies could be the examination and comparison with check point inhibitor therapy in organotypic HNSCC co-cultures.

## 5. Conclusions

In summary, NOTCH1 inhibition impedes migration and proliferation of HNSCC in vitro. Correspondingly, high protein expression of NICD was significantly associated with lower OS in an OPSCC cohort. NICD expression was not associated with tumor infiltrating lymphocytes, but the combination of the immunoscore and NICD expression stratified patients more precisely into different clinical risk groups and could, therefore, be used as a prognostic tool. The targeting of NOTCH1 could be an important therapeutic option in the future, especially in high-risk HNSCC with a low immunoscore.

## Figures and Tables

**Table 1:** Primers and annealing temperatures for qPCR.

Primer	Sequence	Annealing Temperature
NOTCH1 forward NOTCH1 reverse	TGAATGGCGGGAAGTGTGAAG GGTTGGGGTCCTGGCATCG	62 °C
GAPDH forward GAPDH reverse	AGCCACATCGCTCAGACA GCCCAATACGACCAAATCC	56 °C

**Table 2:** Depiction of clinicopathological characteristics and distribution of parameters in high and low NICD or NOTCH1 expression groups in the 70 oropharyngeal squamous cell carcinoma (OPSCC) patients included in this study (60 of these with mRNA data).

Variate	Overall	NICD IHC Expression		*p* Value (Fisher’s Exact)	Overall	NOTCH1 mRNA Expression		*p* Value (Fisher’s Exact)
		Low	High			Low	High	
**Tumor stage (pathological)**								
T1/T2	48 (68.6%)	23	25		43 (71.7%)	26	17	
T3/T4	22 (31.4%)	5	17	0.0659	17 (28.3%)	4	13	0.0204
**Nodal stage (pathological)**								
N0	20 (28.6%)	12	8		17 (28.3%)	10	7	
N1-3	50 (71.4%)	16	34	0.0571	43 (71.7%)	20	23	0.5676
**Metastasis (initial stage)**								
M0	67 (95.7%)	28	39		57 (95%)	29	28	
M1	3 (4.3%)	0	3	0.2696	3 (5%)	1	2	>0.9999
**p16 Status**								
p16 positive	26 (37.1%)	6	20		23 (38.3%)	8	15	
p16 negative	44 (62.9%)	22	22	0.0425	37 (61.7%)	22	15	0.1102
**Gender**								
Male	56 (80%)	24	31		47 (78.3%)	24	23	
Female	14 (20%)	4	11	0.3730	13 (21.7%)	6	7	>0.9999

## Data Availability

The data presented in this study are available in the Supplementary Materials.

## Supplementary Materials

The following supporting information can be downloaded at: https://www.mdpi.com/article/10.3390/cancers14041080/s1, Figure S1: (a) Results of the western blots confirming the knockdown of NOTCH1 in the HNSCC cell lines. (b) OS of OPSCC patients in relation to NOTCH1 mRNA expression (*p* value (log-rank) = 0.706). (c) OS of OPSCC patients in relation to NOTCH1 mRNA expression and the immunoscore (*p* value (log-rank) = 0.152). (d) OS of HPV positive OPSCC patients in relation to NICD expression (*p* value (log-rank) = 0.286). (e) OS of HPV negative OPSCC patients in relation to NICD expression (*p* value (log-rank) = 0.0508). (f) Association of NOTCH1 mRNA expression and NICD expression (Fishers exact test *p* = 0.1923). (g) Association of immunoscore and p16 (Fishers exact test *p* = 0.0406). (h) Median number of T lymphocytes in the areas of the tumor. (i) Exemplary images of immunohistochemical CD3 (left) and CD8 (right) positive T lymphocytes in OPSCC.; File S1: Full Western blot images; Table S1: Detailed Results of (a) migration assay (scratch assay, 9 h) and (b) proliferation assay (72 h) after siRNA mediated NOTCH1 knockdown, inhibition of NOTCH1 signaling via DAPT and stimulation with DLL4.
