# Supplementary material for: NOTCH1 Intracellular Domain and the Tumor Microenvironment as Prognostic Markers in HNSCC
Source: Cancers (Basel). 2022 Feb 21;14(4):1080. doi: 10.3390/cancers14041080 (PMC8870336; doi:10.3390/cancers14041080)
Supplement: Supplementary file 1 [file cancers-14-01080-s001.zip › cancers-1567625-supplementary.pdf]

cancers

MDPI

(a)

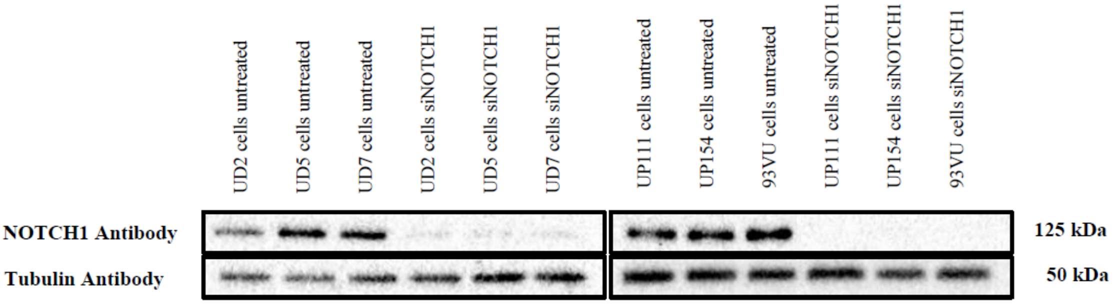

(b)

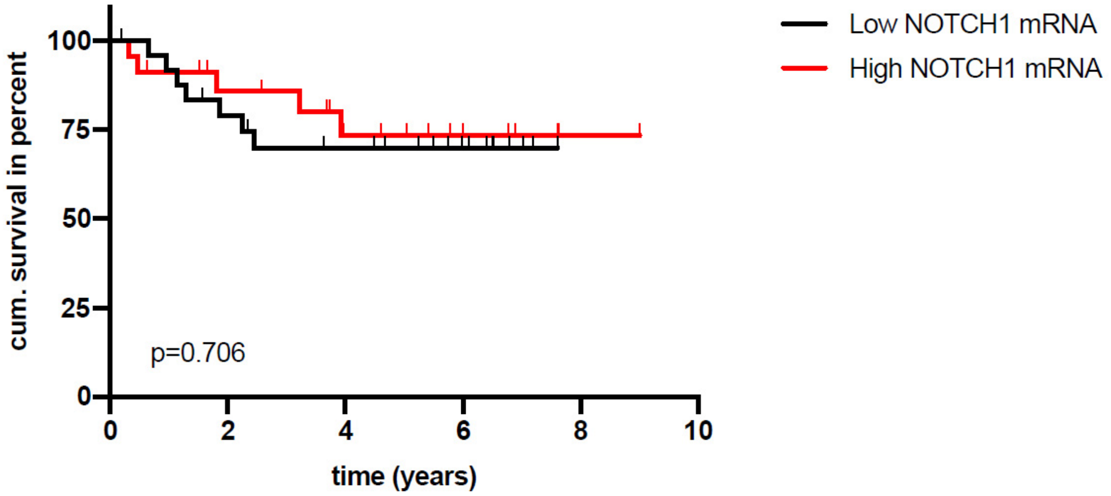

(c)

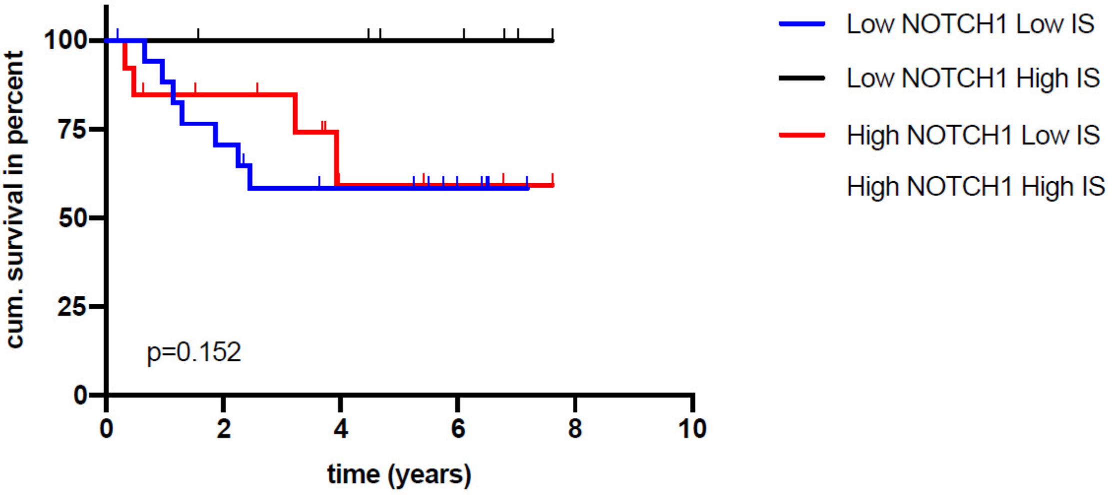

(d)

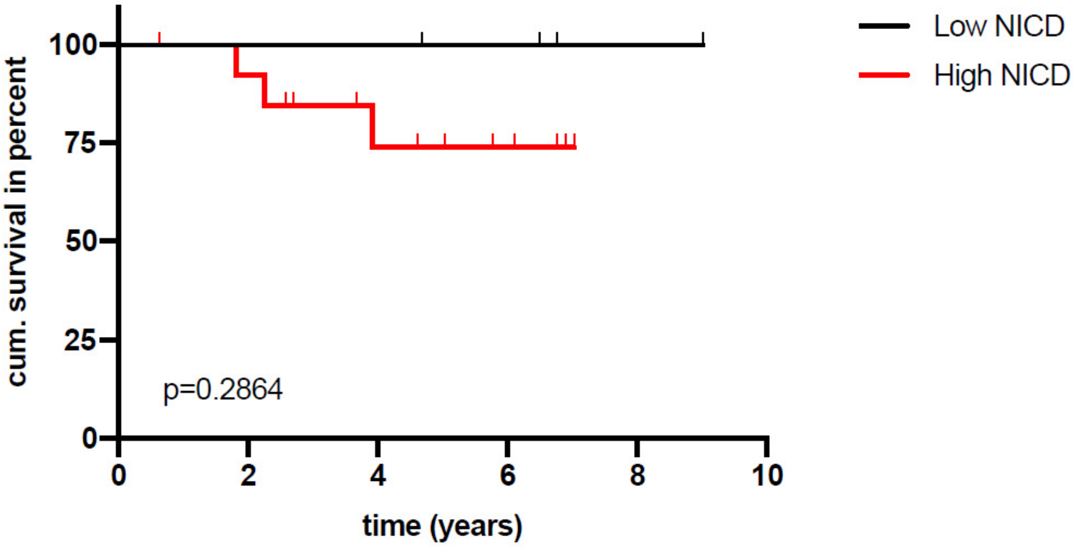

(e)

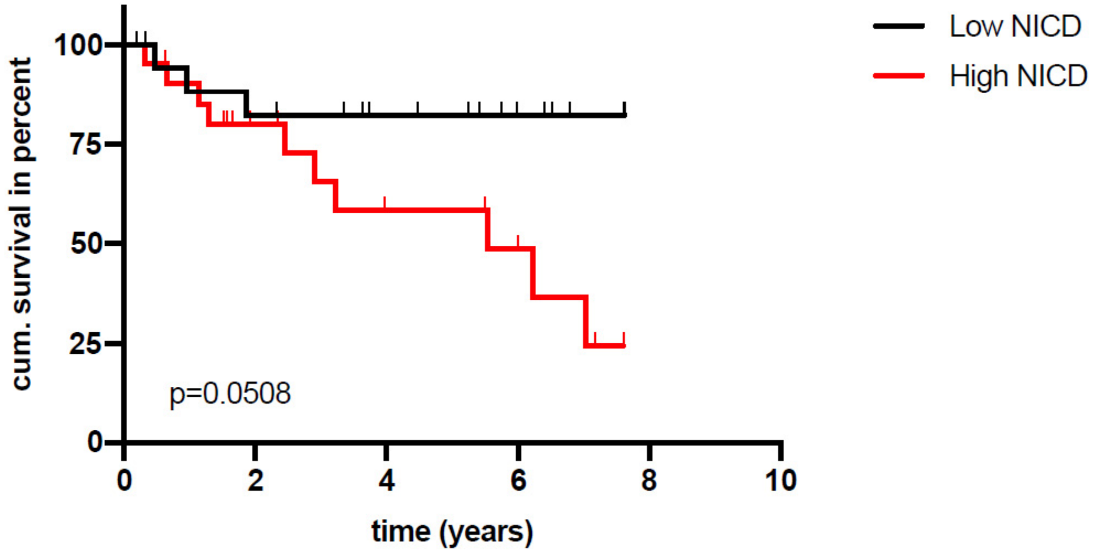

(f)

		NOTCH1 mRNA expression		
		Low	High	
NICD Expression	Low	16	10	
	High	14	20	
				p=0.1923

(g)

		immunoscore		
		Low	High	
p16	Negative	33	11	
	Positive	13	13	
				**p=0.0406**

(h)

	CD3		CD8	
Tumor area	CT	IM	CT	IM
Median number of T lymphocytes per 250 µm^2	77.5	205	60	136.25

(i)

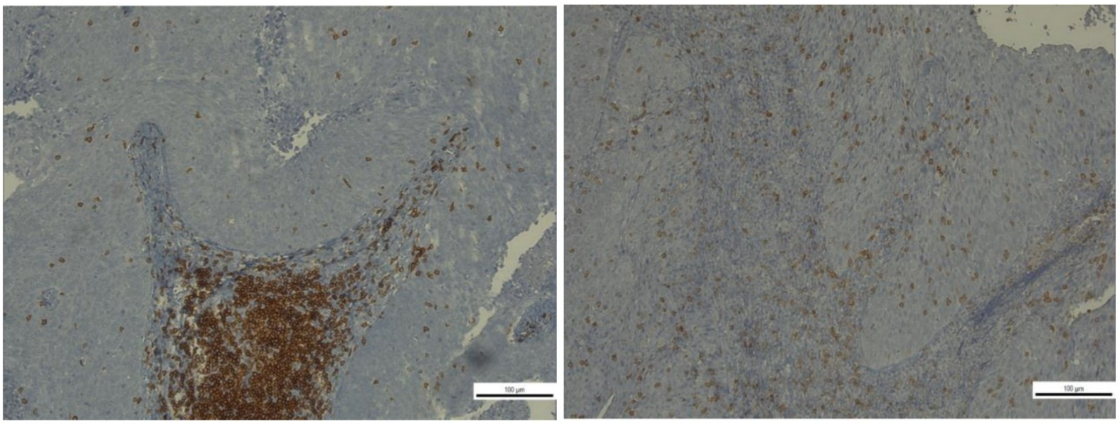

Figure S1. (a) Results of the western blots confirming the knockdown of NOTCH1 in the HNSCC cell lines. On the left side are three cell lines before and 48h after treatment with siRNA targeting NOTCH1 (siNOTCH1) depicted. On the right side the other three cell lines and corresponding knockdown are shown. Tubulin was used as a control. The band size of Tubulin was approximately 50 kDa, the band size of NOTCH1 was approximately 125 kDa. **(b)** OS of OPSCC patients in relation to NOTCH1 mRNA expression (p value (log-rank) =0.706). **(c)** OS of OPSCC patients in relation to NOTCH1 mRNA expression and the immunoscore (p value (log-rank) =0.152). **(d)** OS of HPV positive OPSCC patients in relation to NICD expression (p value (log-rank) =0.286). **(e)** OS of HPV negative OPSCC patients in relation to NICD expression (p value (log-rank) =0.0508). **(f)** Association of NOTCH1 mRNA expression and NICD expression (Fishers exact test p=0.1923). **(g)** Association of immunoscore and p16 (Fishers exact test p=0.0406). **(h)** Median number of T lymphocytes in the areas of the tumor. CT = Core of tumor, IM = Invasive margin. These numbers were used to calculate the immunoscore. **(i)** Exemplary images of immunohistochemical CD3 (left) and CD8 (right) positive T lymphocytes in OPSCC.

Table S1. Detailed Results of (a) migration assay (scratch assay, 9h) and (b) proliferation assay (72h) after siRNA mediated NOTCH1 knockdown, inhibition of NOTCH1 signaling via DAPT and stimulation with DLL4; Depicted are results of all 6 cell lines, the 3 HPV+ cell lines and the 3 HPV- cell lines. Data is depicted as mean ± SD. Significance was tested with one-way ANOVA and Dunnet's multiple testing.

(a)

	Ordinary one-way Anova (p value)	Untreated	NOTCH1 KD	p value (Dunnet's)	DAPT	p value (Dunnet's)	DLL4	p value (Dunnet's)
UD2	0.0784	1	0.341	0.0736	0.561	0.2623	0.978	0.9994
UD5	0.0241	1	0.313	0.0179	0.416	0.0392	0.769	0.5199
UD7	0.0102	1	0.310	0.0042	0.743	0.2660	0.776	0.3572
UP111	0.0734	1	0.528	0.0381	0.757	0.3277	0.877	0.7691
UP154	0.0303	1	0.661	0.0148	0.921	0.6716	0.832	0.2264
93VU	0.0098	1	0.502	0.0314	0.901	0.8604	1.232	0.3661
All cell lines (grouped)	<0.0001	1	0.443	<0.0001	0.715	0.0010	0.911	0.5011
HPV+ cell lines	0.0002	1	0.501	0.0003	0.792	0.1706	1.014	0.9985
HPV- cell lines	<0.0001	1	0.384	<0.0001	0.639	0.0019	0.806	0.1312

(b)

	Ordinary one-way Anova (p value)	Untreated	NOTCH1 KD	p value (Dunnet's)	DAPT	p value (Dunnet's)	DLL4	p value (Dunnet's)
UD2	0.0985	1	0.493	0.2066	0.826	0.8500	1.259	0.6572
UD5	0.0263	1	0.458	0.0861	1.103	0.9317	1.271	0.4897
UD7	0.1589	1	0.101	0.1662	0.855	0.9739	1.135	0.9786
UP111	0.0004	1	0.523	0.0059	0.581	0.0124	1.245	0.1407
UP154	0.2231	1	0.509	0.1818	0.919	0.9761	0.989	>0.9999
93VU	0.1295	1	0.308	0.0931	0.784	0.7869	0.958	0.9974
All cell lines (grouped)	<0.0001	1	0.398	<0.0001	0.845	0.3175	1.143	0.3828
HPV+ cell lines	0.0003	1	0.435	0.0009	0.843	0.5445	1.069	0.9266
HPV- cell lines	<0.0001	1	0.360	0.0011	0.846	0.7193	1.217	0.4609
